# The joint memory effect: challenging the selfish stigma in Huntington’s disease?

**DOI:** 10.1093/braincomms/fcae440

**Published:** 2024-12-09

**Authors:** Romain Dalléry, Nicolas Fraisse, Laurent Cléret de Langavant, Katia Youssov, Graça Morgado, Renaud Massart, Robin Schubert, Ralf Reilmann, Charlotte Jacquemot, Blanche Bapst, Monica Busse, David Craufurd, Anne Rosser, Marine Lunven, Anne-Catherine Bachoud-Lévi

**Affiliations:** Département d’Etudes Cognitives, École normale supérieure, PSL University, 75005 Paris, France; Institut Mondor de Recherche Biomédicale, Equipe NeuroPsychologie Interventionnelle, University Paris Est Creteil, INSERM U955, F-94010 Creteil, France; Service de Neurologie, AP-HP Hôpital Henri Mondor-Albert Chenevier, Centre de référence Maladie de Huntington, F-94010 Créteil, France; NeurATRIS, Hôpital Henri Mondor, 94010 Créteil, France; Département d’Etudes Cognitives, École normale supérieure, PSL University, 75005 Paris, France; Institut Mondor de Recherche Biomédicale, Equipe NeuroPsychologie Interventionnelle, University Paris Est Creteil, INSERM U955, F-94010 Creteil, France; Service de Neurologie, AP-HP Hôpital Henri Mondor-Albert Chenevier, Centre de référence Maladie de Huntington, F-94010 Créteil, France; NeurATRIS, Hôpital Henri Mondor, 94010 Créteil, France; Département d’Etudes Cognitives, École normale supérieure, PSL University, 75005 Paris, France; Institut Mondor de Recherche Biomédicale, Equipe NeuroPsychologie Interventionnelle, University Paris Est Creteil, INSERM U955, F-94010 Creteil, France; Service de Neurologie, AP-HP Hôpital Henri Mondor-Albert Chenevier, Centre de référence Maladie de Huntington, F-94010 Créteil, France; NeurATRIS, Hôpital Henri Mondor, 94010 Créteil, France; Département d’Etudes Cognitives, École normale supérieure, PSL University, 75005 Paris, France; Institut Mondor de Recherche Biomédicale, Equipe NeuroPsychologie Interventionnelle, University Paris Est Creteil, INSERM U955, F-94010 Creteil, France; Service de Neurologie, AP-HP Hôpital Henri Mondor-Albert Chenevier, Centre de référence Maladie de Huntington, F-94010 Créteil, France; NeurATRIS, Hôpital Henri Mondor, 94010 Créteil, France; Inserm, Centre d’Investigation Clinique 1430, AP-HP Hôpital Henri Mondor, 94010 Créteil, France; Inserm, Centre d’Investigation Clinique 1430, AP-HP Hôpital Henri Mondor, 94010 Créteil, France; Département d’Etudes Cognitives, École normale supérieure, PSL University, 75005 Paris, France; Institut Mondor de Recherche Biomédicale, Equipe NeuroPsychologie Interventionnelle, University Paris Est Creteil, INSERM U955, F-94010 Creteil, France; NeurATRIS, Hôpital Henri Mondor, 94010 Créteil, France; R&D-Campus, Technology-Park, George-Huntington-Institute, 48149 Muenster, Germany; R&D-Campus, Technology-Park, George-Huntington-Institute, 48149 Muenster, Germany; Department of Neurodegeneration, Hertie Institute for Clinical Brain Research, University of Tuebingen, 72076 Tuebingen, Germany; Department of Clinical Radiology, University of Muenster, 48149 Muenster, Germany; Département d’Etudes Cognitives, École normale supérieure, PSL University, 75005 Paris, France; Institut Mondor de Recherche Biomédicale, Equipe NeuroPsychologie Interventionnelle, University Paris Est Creteil, INSERM U955, F-94010 Creteil, France; Service de Neurologie, AP-HP Hôpital Henri Mondor-Albert Chenevier, Centre de référence Maladie de Huntington, F-94010 Créteil, France; NeurATRIS, Hôpital Henri Mondor, 94010 Créteil, France; Institut Mondor de Recherche Biomédicale, Equipe NeuroPsychologie Interventionnelle, University Paris Est Creteil, INSERM U955, F-94010 Creteil, France; Service de Neuroradiologie, AP-HP Hôpital Henri Mondor-Albert Chenevier, F-94010 Créteil, France; Centre for Trials Research, Cardiff University, Cardiff CF14 4EP, UK; Wales Brain Research And Intracranial Neurotherapeutics (BRAIN) Biomedical Research Unit, College of Biomedical and Life Sciences, Cardiff University, Cardiff CF14 4EP, UK; Manchester Centre for Genomic Medicine, Manchester Academic Health Science Centre, St Mary’s Hospital, Manchester University NHS Foundation Trust, Manchester M13 9PL, UK; Division of Evolution and Genomic Sciences, Faculty of Biology, Medicine and Health, School of Biological Sciences, Manchester Academic Health Science Centre, University of Manchester, Manchester M13 9PL, UK; Wales Brain Research And Intracranial Neurotherapeutics (BRAIN) Biomedical Research Unit, College of Biomedical and Life Sciences, Cardiff University, Cardiff CF14 4EP, UK; Cardiff University Brain Repair Group, School of Biosciences, Life Sciences Building, Cardiff CF10 3AX, UK; Cardiff School of Medicine, Neuroscience and Mental Health Institute, Hadyn Ellis Building, Cardiff CF24 4HQ, UK; Département d’Etudes Cognitives, École normale supérieure, PSL University, 75005 Paris, France; Institut Mondor de Recherche Biomédicale, Equipe NeuroPsychologie Interventionnelle, University Paris Est Creteil, INSERM U955, F-94010 Creteil, France; Service de Neurologie, AP-HP Hôpital Henri Mondor-Albert Chenevier, Centre de référence Maladie de Huntington, F-94010 Créteil, France; NeurATRIS, Hôpital Henri Mondor, 94010 Créteil, France; Département d’Etudes Cognitives, École normale supérieure, PSL University, 75005 Paris, France; Institut Mondor de Recherche Biomédicale, Equipe NeuroPsychologie Interventionnelle, University Paris Est Creteil, INSERM U955, F-94010 Creteil, France; Service de Neurologie, AP-HP Hôpital Henri Mondor-Albert Chenevier, Centre de référence Maladie de Huntington, F-94010 Créteil, France; NeurATRIS, Hôpital Henri Mondor, 94010 Créteil, France; Inserm, Centre d’Investigation Clinique 1430, AP-HP Hôpital Henri Mondor, 94010 Créteil, France

**Keywords:** Huntington’s disease, joint memory effect, passive cooperation, social cognition

## Abstract

The prevalent belief that individuals with Huntington’s disease exhibit selfish behaviour, disregarding the thoughts, feelings and actions of others, has been challenged by patient organizations and clinical experts. To further investigate this issue and study whether participants with Huntington’s disease can pay attention to others, a joint memory task was carried out in patients with Huntington’s disease with and without a partner. This study involved 69 participants at an early stage of Huntington’s disease and 56 healthy controls from the UK, France and Germany, who participated in the international Repair-HD multicentre study (NCT03119246). Participants completed a semantic categorization task across three categories: animals, fruits and vegetables and manufactured objects. They performed the task either alone (Alone condition) or with the examiner acting as a partner (Pair condition). In the Pair condition, the participant was assigned one category, their partner was assigned another and one category was left unassigned. Afterwards, participants engaged in a surprise free recall task to remember as many words as possible. Words not assigned to anyone were considered socially irrelevant in contrast to the ones assigned to the participant and to the partner. Both groups demonstrated the expected self-prioritization effect, recalling their assigned words better than their partner’s or unassigned words in both conditions. Additionally, a joint memory effect was observed, with better recall for the partner’s assigned words than the unassigned words in the Pair condition (controls: difference = 0.45, *P* < 0.001; participants with Huntington’s disease: difference = 0.34, *P* < 0.001). Socially relevant words were thus better recalled than irrelevant words. The number of recalled words correlated with cognitive performance (all *P*-values < 0.05) and MRI analysis revealed a negative correlation between the joint memory effect and right orbitofrontal grey matter density in participants with Huntington’s disease. These findings challenge the notion that individuals with Huntington’s disease display selfish behaviours because of disinterest in others. They show the ability to process information about their partners, implying that their social difficulties may arise from factors other than social cognition deficits. This opens the door for more ecological assessments of social cognition in patients with Huntington’s disease.

## Teaser

Dalléry *et al*. report that patients with Huntington’s disease, like healthy controls, spontaneously integrate human-relevant information (themselves and their partners) better than human-irrelevant information. These results contradict the hypothesis of selfish behaviour inherent in Huntington’s disease and suggest that patients’ difficulties in social interaction stem from their cognitive limitations.

## Graphical Abstract

**Graphical Abstract:**
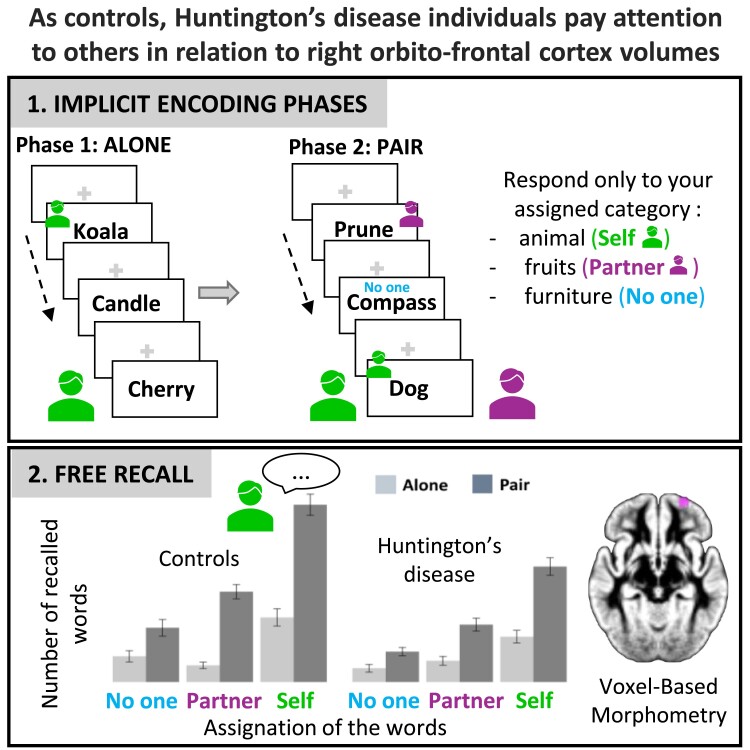

See Stock, Cypers, De Winter and Vandenbulcke (https://doi.org/10.1093/braincomms/fcae462) for a scientific commentary on this article.

## Introduction

Huntington’s disease is an autosomal dominant disorder of the CNS caused by a CAG repeat expansion in the huntingtin gene.^1^ Huntington’s disease usually manifests in the fourth or fifth decade of life with progressive uncontrollable movements, cognitive deterioration and neuropsychiatric symptoms.^2^ Huntington’s disease continues to be one of the most stigmatised diseases globally impacting the physical, emotional, cognitive and social spheres of patients and their relatives.^3^ One major stereotype among the many factors contributing to stigma is that individuals with Huntington’s disease can frequently exhibit selfish behaviours and do not pay attention to others. This stigma is reflected in various literary works, blog articles and family discussions. For instance, Wallace wrote in 1972: ‘There is clinically […] gross emotional disturbance with the characteristic extraordinarily selfish egocentric personality of the typical Huntington’s choreic’.^4^ An insurance company’s description of the disease further highlights this entrenched view: ‘Your personality can gradually become more self-centred and unmotivated, putting a strain on personal relationships’.^2^ However, whilst this view has garnered criticism from some authors^5^ and patients’ lay associations, there remain suggestions in a variety of settings that patients report difficulties in perspective taking yielding to ‘egocentric perspective, behavioural inflexibility, socially inappropriate behaviour and lack of insight’,^6^ thus emphasizing the selfish behaviour stigma. Here, to refute such stigma, we assessed the capacity of patients with Huntington’s disease to spontaneously pay attention to others.

The limited understanding of the mechanisms behind these reported selfish behaviours is a critical consideration. Selfishness is a multifaceted construct characterized by a focus on one’s own interest, desires and well-being at the expense of, or without due consideration for the interests, needs or well-being of others.^7,8^ It can manifest as a moral stance that prioritizes self-interest,^9^ a psychological trait to primarily serve one own’s needs,^7^ behaviours that neglect or disregard the welfare of others^10^ and social dynamics undermining relationships and cooperation.^11^ While the concept of selfishness is rather intuitive, it cannot be evaluated in all its facets. Whatever the chosen perspective, selfishness implies either a lack of attention or insufficient attention to others compared with the one dedicated to the self. This suggests that the question of selfishness in these patients could be tackled by assessing whether patients with Huntington’s disease differ from healthy participants regarding the way in which they balance their attention between others and themselves.

One way of appraising the social interactions of patients with Huntington’s disease is through social cognition tasks, such as theory-of-mind (ToM) tasks. ToM tasks are designed to assess the ability to comprehend one’s own and others’ mental states, including other people’s intentions, motivations, beliefs and emotions. It is well established that patients with Huntington’s disease display deficits in performance in these tasks in comparison with controls.^12-14^ Impairments in ToM support the hypothesis that individuals with Huntington’s disease may have difficulty attributing mental states to others or ‘putting themselves in the shoes of others’. Allain *et al.*’s^15^ study, for example, demonstrated that patients with Huntington’s disease failed to identify the thoughts of cartoon characters in social situation and thus correctly attribute them intention when compared with controls. These findings were later confirmed by other studies using the same, or very similar tasks, which were analysed in a meta-analysis in 2016.^14^ Such task may, however, inherently present certain pitfalls and does not truly allow for the study of the notion of selfishness. First, because of the nature of the task, one cannot exclude that performance of patients with Huntington’s disease at the intention attribution tasks may be impacted by their attentional and executive deficits.^16-18^ Second, regarding the connection to selfishness, while cognitive ToM tasks involve paying attention to others, they do not predict anything regarding the balance between attention to the self and attention to other. In other words, one could perfectly infer someone else feelings without taking any account of those feelings.

Another approach has been to explore the embodied cognition capacities of individuals with Huntington’s disease as a means of gaining insight into their social interactions. In this theory, cognitive processes are deeply rooted in the body’s interactions with the world.^19^ Applied to emotions, it suggests that perceiving others’ emotional states requires sensorimotor reexperiencing rather than relying solely on abstract conceptual representations of emotions.^20^ Presymptomatic gene carriers of the Htt gene experience abnormal bodily sensations during anger^21^ as well as low awareness of emotions and internal body states.^22^ Facial expressions and autonomic response to negative emotions are also impaired in patients with Huntington’s disease.^23^ It has been suggested that deficits in embodied cognition may impede not only their recognition of emotional states, but also their behaviour through emotional dysregulation and increased irritability.^22^ This in turn could result in a lack of interest in the impact of own’s feelings on others, which could be akin to selfishness.

Besides behavioural data, exploring brain regions associated with selfish behaviours could help discern which theory between ToM and embodied cognition better explains such behaviours. Key ToM regions include the anterior paracingulate cortex, posterior superior temporal sulcus and temporal pole.^24^ The orbitofrontal cortex has also been implicated, especially in considering others’ emotions.^25,26^ Key brain regions implicated in embodied cognition include the posterior superior temporal sulcus, posterior parietal cortex, anterior insula, amygdala and premotor cortices.^27^

From our perspective, one significant limit of ToM and embodied cognition tasks is their detachment from real-life contexts, questioning their ability to fully reflect the patient’s social cognition skills and capacity to pay attention to the self and to others. In these tasks, participants are often isolated and tested with computers, devoid of social interactions where a physical presence of a social partner plays a crucial role.^28^ In the light of these limitations, we opted to utilize laboratory settings of joint tasks which are real-time dual interaction paradigms requiring mutual understanding and passive cooperation.^29^ This avoids interference with the classical concepts of social interaction to adapt them to that of selfishness by establishing an artificial link between the concepts they convey and the gradient of interest between oneself and others explicit in selfishness.

Whereas joint paradigms have been tested in a variety of cognitive functions such as attention^30^ or memory performance,^31-33^ here we evaluate whether individuals with Huntington’s disease spontaneously pay attention to others and to themselves, as a premise of exploring selfish behaviours. In these tasks,^31-33^ healthy participants are classically biased towards memorization of their own target objects or items [self-prioritization effect (SPE)],^34^ but objects that are the targets of others’ actions also receive enhanced encoding compared with the ones attributed to the computer (or to no one as a control condition). This result, known as the joint memory effect (JME), implies that the presence of others influences our basic memory for objects and events in our environment.^35^

Considering that the difficulties of patients with Huntington’s disease in representing their own body movements as well of body movement of a partner impact their capacity to grasp their own and others feelings,^21,22^ one could expect that both JME and SPE would decreased. In contrast, selfishness would imply an increase of SPE and a decrease of JME compared with controls. We also tested how cognitive and psychiatric features could be related to SPE and JME and complemented our behavioural experiment with morphometrical MRI to assess whether ToM and embodied cognition brain networks were impacted in our task.

## Materials and methods

### Participants

Sixty-nine patients with Huntington’s disease with at least 38 CAG repeats^36^ in the mutant Htt gene of Huntington’s disease and at an early stage of the disease and 56 healthy controls were recruited from four sites: Créteil, France (*n* = 39 participants with Huntington’s disease and 34 controls), Muenster, Germany (*n* = 22 participants with Huntington’s disease and 19 controls), Manchester, UK (*n* = 4 participants with Huntington’s disease and 3 controls), and Cardiff, UK (*n* = 4 participants with Huntington’s disease and 0 control) in the CAPIT-HD study NCT 03119246 [Beta Testing of a New Assessment in Huntington’s Disease (HD)] in the framework of the European REPAIR-HD project (https://cordis.europa.eu/project/id/602245/reporting/fr), which aimed to establish assessments for novel therapies. Forty-nine Htt gene carriers were at Stage I and 28 at Stage II according to the Total Functional Capacity score.^37^ Healthy controls participants were matched to the patients’ age, sex and level of education (Table 1). Controls were included with a Mattis Dementia Rating Scale^38^ total score of more than 136 and no alcohol or substance abuse, nor neurological comorbidity. The study was approved by the French Research Ethics Committee (CPP Ile de France III). All participants gave written informed consent before participating in the study.

**Table 1:** Demographics, neurological description and general assessment for controls and participants with Huntington’s disease

	Controls	Participants with Huntington’s disease	*P*-value
*N*	56	69	–
(Cardiff/Créteil/Manchester/Muenster)	(0/34/3/19)	(4/39/4/22)	–
Handedness^a^	2A/47R/7L	63R/6L	0.21
Gender^b^	30F/26M	24F/45M	0.054
Age (years)	51.74 ± 9.98 (32− 70)	52.05 ± 10.41 (23− 73)	0.86
Education (years)	13.91 ± 3.24 (8− 24)	14.54 ± 2.96 (9− 20)	0.26
*N* CAG^c^ repeats	–	43.75 ± 3.15 (38− 55)	–
TFC^d^	**13.00 ± 0** (**13− 13**)	**10.72 ± 1.68** (**7− 13**)	**<0.0001**
TMS^e^	**0.68 ± 1.2** (**0− 6**)	**28.61 ± 14.8** (**1− 60**)	**<0.0001**
Disease burden score^f^	–	402.8 ± 80.9 (134.7− 601.3)	**–**

Unless otherwise specified, quantitative values are means ± standard deviations and range (). Significant difference between groups are highlighted in bold.

^a^A ambidextrous, L left, R right.

^b^F female, M male.

^c^ *N* CAG repeats: number of cytosine adenine guanine triplets repeats.

^d^TFC, Total Functional Capacity.

^e^TMS, Total Motor Score.

^f^Disease burden score = Age × (CAG repeat – 35.5).

### General assessment

Participants were evaluated with the Total Functional Capacity (TFC), the Total Motor Score (TMS), the Letter Verbal Fluency over 1 min, the Symbol Digit Modalities Test (SDMT) and the Stroop tests (Colour, Word and Interference) from the Unified Huntington’s Disease Rating Scale (UHDRS^®^).^39^ Additional evaluations were undertaken: the short version of Problem Behaviours Assessment,^40^ the Mattis Dementia Rating Scale,^38^ the Categorical Fluency Task over 1 min and the Hopkins Verbal Learning Memory Task.^41,42^ The overall disease severity was assessed with the composite Unified Huntington’s Disease Rating Scale (cUHDRS) score.^43^


$$\begin{array}{rl} \mathrm{cUHDRS}\;= & [\left(\frac{\mathrm{TFC}-10.4}{1.9}\right)-\left(\frac{\mathrm{TMS}-29.7}{14.9}\right)\\ & \;+\left(\frac{\mathrm{SDMT}-28.4}{11.3}\right)+\left(\frac{\mathrm{Stroop}\;\mathrm{Word}-66.1}{20.1}\right)]\\ & +10 \end{array}$$

### Joint memory task

**Figure 1:**
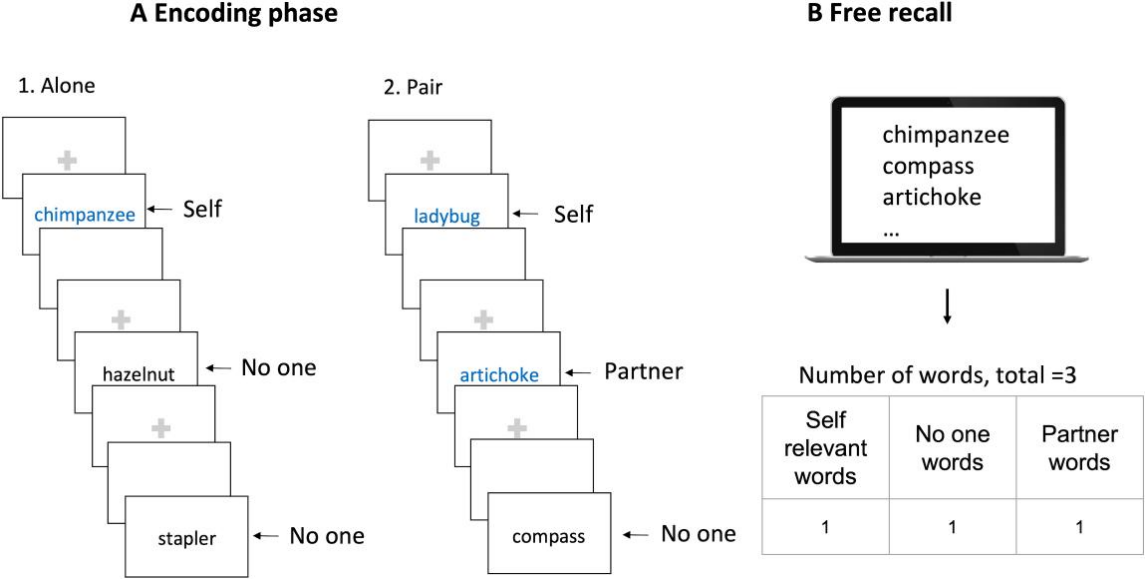
**Design of the joint memory task.** Encoding phase (**A**): in the first part, participants worked alone (Alone condition) and then with the examiner acting as a partner (Pair condition) on the semantic categorization task. Surprise free recall phase (**B**): participants were asked to recall as many words as they could, regardless of the categories of the words.

The joint memory task is adapted from the procedure described in Eskenazi *et al*.^33^ The task relies on the recall of words of three categories, namely animals, fruits and vegetables and manufactured objects. One of these three categories was assigned either to the participant, to the examiner who acted as a partner or to no one as a control condition. The experiment comprised two parts (detailed below): (i) a semantic categorization task (including two conditions: assessment of the participant alone followed by the assessment of the participant together with the partner, in pair) and (ii) a free recall task (Fig. 1). The semantic categorization task allows a passive encoding of the words, whereas the free recall task allows the comparison of the number of words recalled in each category to determine whether the participant had paid attention to the assessment of the partner.

### Materials

We selected 96 words distributed in three lists of 32 words belonging to each semantic category. The three lists of words were matched for syllable length (the mean syllable number of animals: 2.5 ± 0.50; fruits and vegetables: 2.5 ± 0.50; manufactured objects: 2.5 ± 0.50; *P* = 1), phonemes number (the mean phoneme number of animals: 4.8 ± 0.8; fruits and vegetables: 5.1 ± 1.3; manufactured objects: 5.0 ± 1.3; *P* = 0.55) and word frequency (the mean word frequency of animals: 5,6 ± 6.4; fruits and vegetables: 6.0 ± 19.8; manufactured objects: 5.6 ± 7.4; *P* = 0.1). This resulted in 16 words per category being displayed in the Alone condition and the remaining 16 in the Pair condition. For the English and German versions, words have been translated from the French list (Supplementary Table 1).

### Procedure

#### Semantic categorization task

At the beginning of the experiment, the sitting position of the participant and partner (right or left facing the screen) was randomly selected. In the Alone condition, participants sat alone in front of the computer screen. In the Pair condition, participants sat next to a partner, on chairs fixed to the left and right sides of a computer and responded using the same keyboard. Participants ran the semantic categorization alone first and then along with the partner (Fig. 1).

One of the three semantic categories was randomly assigned to the participant, which was labelled ‘self-words’. Another category was assigned to the partner, which was labelled ‘partner-words’. The third was left unassigned, meaning that neither the participant nor the partner had to respond to words from that category, which was labelled ‘no-one words’ (Supplementary Table 1). This allowed us to contrast the categories based on their social value. Words were labelled ‘socially relevant’ when they were attributed to the participants with Huntington’s disease (self-words) or to the partner (partner-words), but socially irrelevant when they were assigned to neither the participant nor the partner (no-one words). Participants were informed of their categories with an oral instruction. The category in the Alone and Pair conditions remained the same for each participant using half of the items list for each condition to control for a recency effect. Each trial began with the presentation of a fixation screen for 1 s, followed by a word stimulus (3 s) and a blank interval (1.5 s) (Fig. 1A). In both Alone and Pair conditions, words were displayed serially on a computer screen. The order in which the words were presented was randomly selected with constraints: (i) the same word was never presented twice in a row and (ii) there were never more than three words of the same semantic category presented one after the other, thus ensuring that participants never waited for more than six turns before being prompted to respond. Participants were instructed to press a key as quickly as possible when a word belonging to their assigned category appeared on the screen (the key ‘Q’ when they sat on the left of the computer or the key ‘M’ when they sat on the right side). They were asked to refrain from responding to words not belonging to their category. In the Pair condition, the partner had to press a key when the word displayed on the screen corresponded to the category assigned to her/him (Fig. 1A). Every time a participant (or the partner) responded to a word by pressing a key, this word, initially written in black, became blue (RGB: 0, 0, 128).

To encourage spontaneous memorization, each word was presented twice, resulting in 16 words from each category being presented twice in each condition. Altogether in the Alone and Pair conditions, the participant was exposed to 192 trials (96 per condition) and pressed the key in the 64 trials corresponding to his/her category if he/she performed the task correctly (32 in the Alone and 32 in the Pair conditions).

The semantic categorization task lasted around 20 min, including instructions to the participant. One-third of the trials required a response from the participant (self-words), one-third of the trials never required a response (no-one words), and one-third did not require any response in the Alone condition but required a response from the partner in the Pair condition (partner-words).

Both word presentation and response recording were performed in Python using the PsychoPy toolbox (https://www.psychopy.org/). Reaction times (from the appearance of the word to the response calculated in ms) and accuracy (percentage of key presses in response to the participant’s assigned category) were recorded.

#### Free recall task

Following the semantic categorization task, participants were instructed to recall as many words as possible, irrespective of their category or condition, without any intervening filler task between the two tests. Participants were not told beforehand that they would be asked to perform a memory task and were allowed to take as much time as necessary (Fig. 1B). Recalled words were labelled either self-words, partner-words or no-one words.

### MRI data acquisition and processing

Three-dimensional T_1_-weighted brain MRI data were collected at 2 centres (Henri Mondor Hospital and Munster) for 42 patients with Huntington’s disease and 39 controls and analysed using the standard processing stream of FSL-VBM^44-46^ (https://fsl.fmrib.ox.ac.uk/fsl/fslwiki/FSLVBM). Details of acquisition parameters and processing were described in Lunven *et al*.^47^ Both groups were matched in age (patients with Huntington’s disease: 51.42 ± 10.62; controls: 51.72 ± 9.90, *P* = 0.90) and education level (patients with Huntington’s disease: 14.73 ± 3.07; controls: 13.85 ± 3.00, *P* = 0.19). There were more males in the Huntington’s disease group than in the controls one (13 females/29 males versus 23 females/16 males, respectively*, P* = 0.02).

## Results

Data management, statistical analyses and graphics generation were conducted with R version 4.0.4 (R Core Team 2020) and RStudio (http://www.rstudio.com). Missing data in cognitive pencil-and-paper tests were imputed using 10 iterations of the non-parametric random forest imputation algorithm. All measures were normalized before statistical comparisons using natural log transformation.

Eleven participants (eight Huntington’s disease including four Huntington’s disease Stage I, four Huntington’s disease Stage II and three controls) were excluded from the cohort due to poor performance on the semantic categorization task, with an accuracy of <80%.^48^ Data from 125 participants were available for the analyses. Patients with Huntington’s disease and controls were matched for age, gender, handedness distributions and education level (Table 1).

### Joint memory task

#### Semantic categorization task

Data are presented in Supplementary Table 2. We performed ANOVAs to compare performance in participants with Huntington’s disease and controls in terms of accuracy and reaction times during the categorization task with group (controls and patients), condition (Alone and Pair) and their interaction as independent factors.

Accuracy in semantic categorization was lower in participants with Huntington’s disease than in controls [*F*(1, 248) = 16.17, *P* < 0.00, *ηp*^2^ = 0.06, 95% confidence interval (CI) (0.02, 1.00)], whatever the condition [*F*(1, 248) = 3.56, *P* = 0.06, *ηp*^2^ = 0.01, 95% CI (0.00, 1.00)] without any interaction between group and condition [*F*(1, 248) < 1].

Response to assigned category was also slower in participants with Huntington’s disease than controls [*F*(1, 248) = 71.83, *P* < 0.001, *ηp*^2^ = 0.22, 95% CI (0.15, 1.00)]. Both groups were faster to target words in the Pair condition than in the Alone condition [*F*(1, 248) = 8.32, *P* = 0.004, *ηp*^2^ = 0.03, 95% CI (6.00e^−03^, 1.00)] without interaction between group and condition [*F*(1, 248) < 1].

#### Free recall task

**Figure 2:**
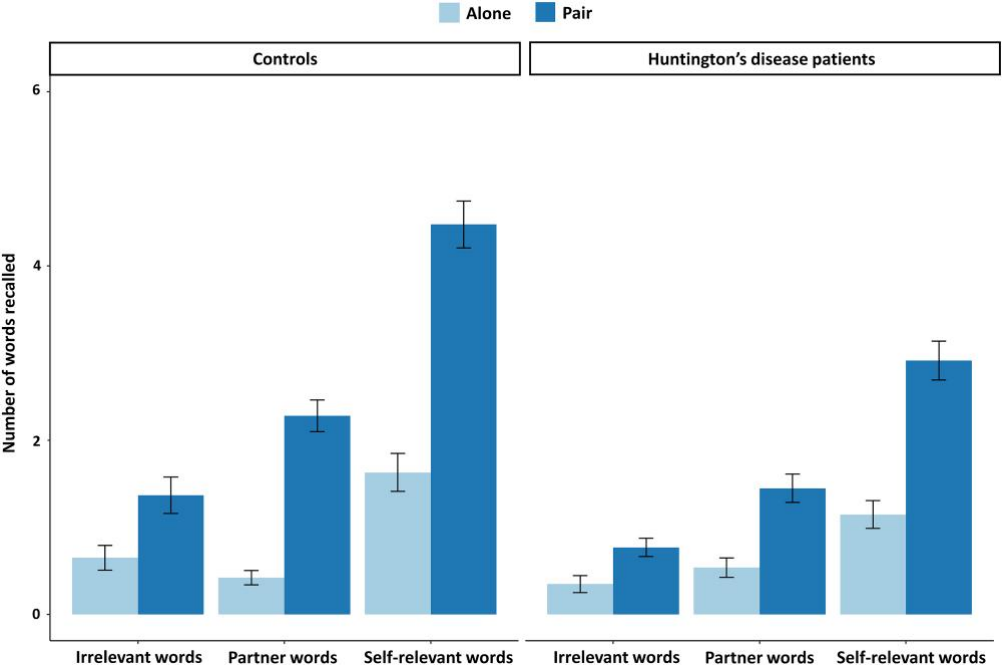
**Presentation of free recall performance.** Illustration of mean number of words recalled during the free recall test, as a function of social value of word (self-relevant words, partner-words and irrelevant words) and conditions (Alone condition versus Pair condition) in participants with Huntington’s disease (*n* = 69) and controls (*n* = 56). Significant interaction between condition and social value in an ANOVA of recalled words with group, social value (self versus irrelevant) and condition [Alone versus Pair: *F*(1, 492) = 42.24, *P* < 0.001] without any other interaction [between condition and group: *F*(1, 492) = 0.18, *P* = 0.67; social value and group: *F*(1, 492) = 3.20, *P* = 0.07; or three-way interaction: *F*(1, 492) = 2.55, *P* = 0.11]. Tukey comparisons showed an SPE in both groups, with more self-words recalled than irrelevant words in both Alone and Pair conditions (Alone: difference = 0.42, *P* < 0.001; Pair: difference = 1.01, *P* < 0.001). Self-words (difference = 0.60, *P* < 0.001) but not irrelevant words (difference = 0.003, *P* = 0.99) were recalled more in the Pair compared with the Alone condition. Significant triple interaction in ANOVA between group, social value (partner versus irrelevant) and condition (Alone versus Pair) [*F*(1, 492) = 4.34, *P* = 0.038]. Tukey *post hoc* analysis confirmed a JME in both groups, with higher recall of partner-words than irrelevant words in the Pair condition (controls: difference = 0.45, *P* < 0.001; participants with Huntington’s disease: difference = 0.34, *P* < 0.001). Recall of partner-words increased in the Pair condition compared with Alone for both groups (controls: difference = 0.51, *P* < 0.001; participants with Huntington’s disease: difference = 0.27, *P* < 0.01), while recall of irrelevant words remained unchanged (controls: difference = −0.06, *P* > 0.99; participants with Huntington’s disease: difference = 0.05, *P* > 0.99).

On average, participants with Huntington’s disease recalled less words than controls (the mean of total recalled words: 7.16 ± 4.30 and 10.98 ± 4.53, respectively, *t*(123) = 4.57, *P* < 0.001). Figure 2 summarizes the recall results by condition and groups. ANOVAs were conducted on the number of recalled words with group (controls and patients), social value (relevant, irrelevant), condition (Alone and Pair) and their interaction as independent factors. This allowed to calculate the SPE by comparing the recall of the self-words to the one of the irrelevant words and the JME by comparing the recall of the partner-words to the one of the irrelevant words.

#### Self-prioritization effect

The first ANOVA was conducted on the number of recalled words with group (controls and patients), social value (self-words and irrelevant words), condition (Alone and Pair) and their interaction as independent factors. Participants with Huntington’s disease recalled less words than controls [*F*(1, 492) = 14.34, *P* < 0.001, *ηp*^2^ = 0.03, 95% CI (9.12e^−03^, 1.00)]. The recall was higher for self-words compared with irrelevant words [*F*(1, 492) = 243.61, *P* < 0.001, *ηp*^2^ = 0.33, 95% CI (0.28, 1.00)] and in the Pair than in the Alone condition: [*F*(1, 492) = 43.02, *P* < 0.001, *ηp*^2^ = 0.08, 95% CI (0.05, 1.00)]. The interaction between condition and social value was significant [Supplementary Fig. 1; *F*(1, 492) = 42.24, *P* < 0.001, *ηp*^2^ = 0.08, 95% CI (0.05, 1.00)], in contrast to the interactions between condition and group, social value and group and the triple interactions which were not significant [*F*(1, 492) = 0.18, *P* = 0.675, *ηp*^2^ = 3.58e^−04^, 95% CI (0.00, 1.00); *F*(1, 492) = 3.20, *P* = 0.07, *ηp*^2^ = 6.45e^−03^, 95% CI (0.00, 1.00), *F*(1, 492) = 2.55, *P* = 0.11, *ηp*^2^ = 5.16e^−03^, 95% CI (0.00, 1.00)]. Tukey comparisons of means confirmed the presence of an SPE in both groups. In the Alone and in the Pair conditions, participants recalled more self-words than irrelevant ones [difference = 0.42, *P* < 0.001, 95% CI (0.25, 0.58); difference = 1.01, *P* < 0.001, 95% CI (0.84, 1.17)]. Participants recalled more self-words in the Pair condition compared with the Alone condition [difference = 0.60, *P* < 0.001, 95% CI (0.43, 0.76)]. This latter difference was not observed for the irrelevant words [difference = 0.003, *P* = 0.99, 95% CI (−0.16, 0.17)].

#### Joint memory effect

A second ANOVA was conducted on the number of recalled words with group (controls and participants with Huntington’s disease), social value (partner-words and irrelevant words) and condition (Alone and Pair) and their interaction as independent factors. Both groups behave similarly [*F*(1, 492) = 2.32, *P* = 0.13, *ηp*^2^ = 4.65e^−03^, 95% CI (0.00, 1.00)]. The recall was higher for partner’s words compared with irrelevant words [*F*(1, 492) = 23.73, *P* < 0.001, *ηp*^2^ = 0.05, 95% CI (0.02, 1.00)] and in the Pair than in the Alone condition [*F*(1, 492) = 22.23, *P* < 0.001, *ηp*^2^ = 0.04, 95% CI (0.02, 1.00)]. The interaction between condition and social value was significant [*F*(1, 492) = 21.60, *P* < 0.001, *ηp*^2^ = 0.04, 95% CI (0.02, 1.00)], but not the ones between condition and group nor between social value and group [*F*(1, 492) = 0.58, *P* = 0.45, *ηp*^2^ = 1.18e^−03^, 95% CI (0.00, 1.00); *F*(1, 492) = 0.52, *P*= 0.47, *ηp*^2^ = 1.06e^−03^, 95% CI (0.00, 1.00)]. The triple interaction was significant [Supplementary Fig. 2; *F*(1, 492) = 4.34, *P* = 0.038, *ηp*^2^ = 8.74e^−03^, 95% CI (2.57e^−04^, 1.00)]. Tukey *post hoc* analysis confirmed the presence of a JME in both groups by a higher recall of partner-words than of irrelevant words in both groups in the Pair condition [controls: difference = 0.45, *P* < 0.001, 95% CI (0.19, 0.71); participants with Huntington’s disease: difference = 0.34, *P* < 0.001, 95% CI (0.10, 0.57)]. The recall of partner-words increased in the Pair condition compared with the Alone condition in both groups [controls: difference = 0.51, *P* < 0.001, 95% CI (0.25, 0.77); participants with Huntington’s disease: difference = 0.27, *P* < 0.01, 95% CI (0.04, 0.51)] in contrast to the recall of irrelevant words that did not changed between conditions [controls: difference = −0.06, *P* > 0.99, 95% CI (−0.32, 0.20); participants with Huntington’s disease: difference = 0.05, *P* > 0.99, 95% CI (−0.18, 0.29)].

### Associations between SPE and JME with general assessment and grey matter volumes

We computed the SPE and the JME scores for the general assessment and MRI analyses. The SPE was defined by the number of self-words recalled minus the number of irrelevant ones, and the JME by the number of partner’s words recalled during the Pair condition minus the number of partner’s words recalled during the Alone condition. First, we conducted ANOVAs on the SPE and JME scores, respectively, with group and total number of recalled word and their interaction as independent factors before running statistical analysis with general assessment and grey matter volumes. The SPE and JME scores were higher in controls than in participants with Huntington’s disease [SPE: controls mean *m* = 4.14, participants with Huntington’s disease *m* = 2.94, *F*(1, 121) = 5.98, *P* < 0.016, *ηp*^2^ = 0.05, 95% CI (4.86^−03^, 1.00)]; JME: controls *m* = 1.91, participants with Huntington’s disease *m* = 0.91, *F*(1, 121) = 18.52, *P* < 0.001, *ηp*^2^ = 0.13, 95% CI (0.05, 1.00)]. The total number of recalled words accounted for the both scores [SPE: *F*(1, 121) = 25.49, *P* < 0.001, *ηp*^2^ = 0.17, 95% CI (0.08, 1.00)]; JME: *F*(1, 121) = 16.92, *P* < 0.001, *ηp*^2^ = 0.12, 95% CI (0.05, 1.00)]. We did not report interaction between group and total number of recalled words [SPE: *F*(1, 121) = 1; JME: *F*(1, 121) < 1]. We found a significant positive Pearson’s correlation between SPE and JME scores (*r* = 0.25, *P* = 0.005).

**Figure 3:**
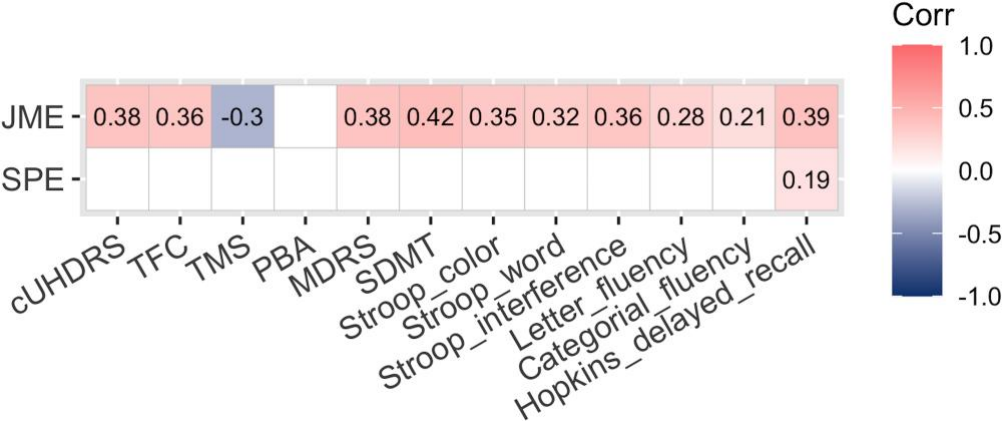
**Spearman’s correlation analysis between the self-prioritization and JMEs and clinical performance in participants with Huntington’s disease (*n* = 69).** Only significant associations are represented by colour after false discovery rate (FDR) correction for multiple tests (FDR adjusted *P*-value <0.05). cUHDRS, composite Unified Huntington’s Disease Rating Scale; DR, delayed recall; MDRS, Mattis Dementia Rating Scale; PBA, short version of Problem Behaviours Assessment; SDMT, Symbol Digit Modalities Test; TFC, Total Functional Capacity; TMS, Total Motor Score.

Scores obtained from the general assessment are summarized in Table 2. Participants with Huntington’s disease performed poorer than controls in all clinical scales (all *P*-values <0.0001). The JME correlated with the performance on the global assessment (Mattis Dementia Rating Scale and cUHDRS) and all cognitive tasks (SDMT, Stroop task, Literal fluency and Hopkins Verbal Learning test) but not on the psychiatric scale (short version of Problem Behaviours Assessment). The SPE only correlated with the Hopkins Verbal Learning test (delayed recall score) (Fig. 3).

**Table 2:** General assessment in controls and participants with Huntington’s disease

General assessments	Controls (*n* = 56)	Patients with Huntington’s disease (*n* = 69)	*P*-value
PBA-s total score^a^	3.95 ± 6.22	10.79 ± 10.33	**<0.0001**
cUHDRS^b^	17.08 ± 1.32	10.55 ± 2.85	**<0**.**0001**
Mattis Dementia Rating Scale^c^	141.9 ± 2.2	133.4 ± 6.8	**<0**.**0001**
Literal fluency (1 min)	41.03 ± 10.01	27.71 ± 9.22	**<0**.**0001**
SDMT^d^	50.82 ± 10.11	30.26 ± 9.4	**<0**.**0001**
STROOP colour	77.11 ± 10.2	49.61 ± 12.8	**<0**.**0001**
STROOP word	101.8 ± 13.8	68.9 ± 16.5	**<0**.**0001**
STROOP interference	44.50 ± 8.89	27.09 ± 8.33	**<0**.**0001**
Hopkins delayed recall	9.98 ± 2.06	5.80 ± 3.02	**<0**.**0001**
Categorical fluency (1 min)	21.93 ± 6.25	14.42 ± 4.72	**<0**.**0001**

Unless otherwise specified, quantitative values are means ± standard deviations. Significant difference between groups are highlighted in bold.

^a^PBA-s total score.

^b^cUHDRS, composite Unified Huntington’s Disease Rating Scale.

^c^MDRS, Mattis Dementia Rating Scale.

^d^SDMT, Symbol Digit Modalities Test.

**Figure 4:**
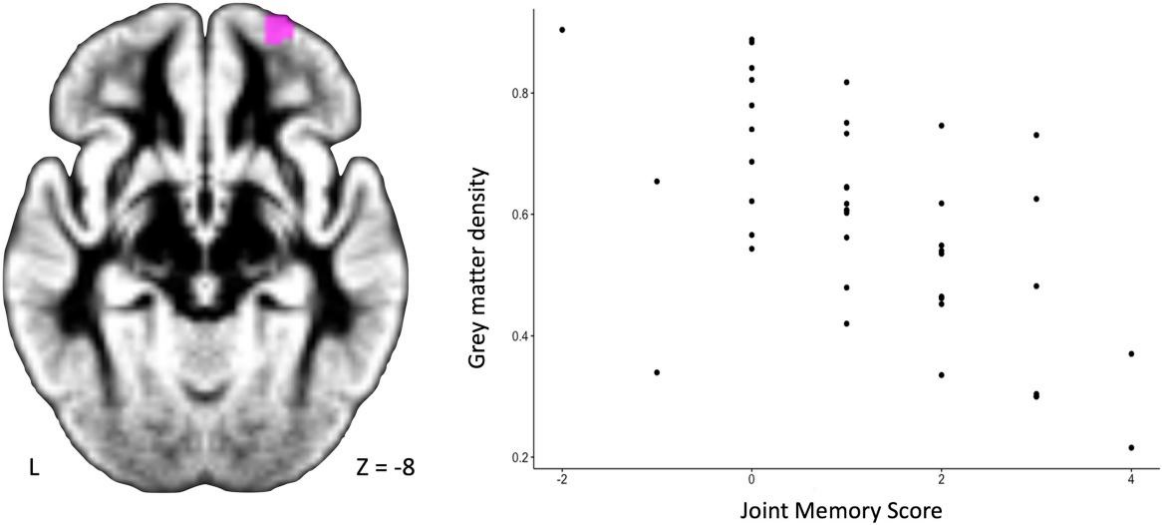
**Voxel-based morphometry MRI analysis in patients with Huntington’s disease (*n* = 42).** Correlation with the JME: the JME was negatively associated with grey matter volumes in the right middle orbital part of the frontal lobe (voxels in rose, cluster size: 58 voxels; coordinates of the cluster peak in Montreal Neurological MNI convention: *x* = 28, *y* = 62, *z* = −8; corrected *P*-value: 0.027). Voxel-based morphometric analysis applying voxel-wise general linear models with non-parametric permutation tests (10 000). Family-wise error correction for multiple comparisons implemented threshold-free cluster enhancement using a significance threshold of *P* < 0.05. Age, sex, total intracranial volume and site were entered as covariates.JME, joint memory effect.

We identified the grey matter areas contributing to the regression models of both SPE and JME using a voxel-based morphometric analysis, by applying voxel-wise general linear models with non-parametric permutation tests (10 000). Family-wise error correction for multiple comparisons was performed, implementing threshold-free cluster enhancement using a significance threshold of *P* < 0.05. Age, sex, total intracranial volume and site were entered as covariates for all imaging analyses. An analysis including participants with Huntington’s disease and controls revealed no association of the JME or SPE scores with grey matter volumes. In participants with Huntington’s disease, the JME score was negatively associated with grey matter volumes in the right middle orbital part of the frontal lobe (Fig. 4). We did not find any significant association between grey matter volumes and the SPE in participants with Huntington’s disease or controls nor with the JME score in controls.

## Discussion

The viewpoint that individuals with Huntington’s disease may manifest selfish behaviours, potentially resulting in disruptions in interpersonal relationships, is controversial.^5^

While it may be tempting to ascribe these behaviours to a diminished interest in others, here we provided evidence to the contrary. Individuals with Huntington’s disease and a control group were assessed in a joint memory task comparing their performance in paying attention to others, across four different sites in three different countries. These results indicated that, despite decreased recall of words for participants with Huntington’s disease compared with controls, both groups displayed an SPE, i.e. they had a better recall of self-relevant words than of irrelevant ones. This effect was higher for words encountered in the Pair condition than in the Alone condition. Similarly, both groups displayed a JME. Within each group, the ability to recall partner’s words, which were responded to by the experimenter (in the Pair condition), was notably superior compared with recall when the partner was absent and did not respond (in the Alone condition). This effect was more pronounced in the control group than in participants with Huntington’s disease.

There was no observed enhancement in recall between the two conditions (Alone and Pair) for the irrelevant words. In brief, participants with Huntington’s disease were able to pay attention to their partners, akin to the controls, albeit to a somewhat lesser extent. They demonstrated a remarkable ability to naturally take into account the partner-relevant information without external prompting.

The social aspect of the JME task has been previously demonstrated in the study by Elekes and Sebanz.^31^ In a similar task, they found that the JME occurred selectively when participants engaged in semantic processing, contrasting with situations where they only attended to the colour of the word. This suggests that this mnesic effect towards others arises specifically when processing information relevant to the partner, indicating a form of unconscious social collaboration. One might question why both those with Huntington’s disease and controls exhibit an SPE if the observed effect is social in nature. The SPE refers to the tendency to prioritize one’s own memories over those of others.^49^ It is a strongly validated effect^50-53^ that denotes an advantage in performance to objects related to the self-concept relative to other objects. This cognitive bias virtually always occurs when the self is activated by the situation and cannot be considered a selfish behaviour. The presence of SPE thus reflects the finding that both patients with Huntington’s disease and controls correctly performed the task, i.e. they did not display any major attentional or mnesic retention issues during the task. Interestingly, this result also suggests that self-referential processes may be relatively spared in Huntington’s disease, despite overall cognitive impairments. It is clinically meaningful because self-referential processes are critical for the sense of self and identity, which loss is a common fear in presymptomatic patients,^54^ where projection identification to disease relatives is common.^55^ This contrasts with Alzheimer’s disease and those with behavioural variant fronto-temporal dementia who were unable to replicate the SPE in a memory task, regardless of performance on neuropsychological tests of episodic memory.^56^

Our findings prompt an exploration of their connection to ToM and embodied cognition theories, which have previously aimed to describe these supposed selfish behaviours. Whereas ToM is a key concept in social interactions, it is not enough to explain selfish behaviours on its own. In fact, one can fully comprehend the emotions of another individual but opt not to focus their attention on them, illustrating selfish behaviour. Indeed, research on children’s ToM abilities and selfish behaviours in economic games like the Ultimatum and Dictator games yields conflicting findings. In these games, one player decides how to divide money with another, who can accept or reject the proposal, resulting in no payoff for both if rejected. While Takagishi *et al*.^57^ showed that higher ToM is related to fairness-related behaviours (higher proposals), Cowell *et al.*^58^ found an inversed correlation showing that higher ToM was related to less sharing, with important size difference, even after accounting for age-related differences. Hence, regardless of their performance in ToM tasks, children’s resource allocation choices may vary based on additional factors such as moral considerations like fairness and justice, societal norms within the group and individual decision-making abilities.^59^ ToM capacities appear as a support for resolving complex situations and reach a decision, but distinguish from the systematic bias towards the self-implied in selfishness. Nevertheless, the joint memory task still shares with the ToM concept that paying attention to other participates to the social interaction. Our imaging findings bring our results into close alignment with ToM. Our morphometric MRI analysis revealed a negative correlation between the right orbitofrontal cortex (Brodmann area 11) and the JME in participants with Huntington’s disease. While this cortical region is not a primary area of ToM, it has been previously associated with impaired emotion recognition.^60^ In another study, the authors identified a correlation between lesions in Brodmann Area 11 and two measures of ToM: a second-order false belief task and perspective-taking items from the interpersonal reactivity index.^61^ The negative correlation observed in our study may indicate an inhibitory process, yet the significance of the finding is not robust enough to justify drawing additional conclusions. As expected, no correlation was detected in the control group, as they were not anticipated to possess any atrophic brain regions.

On the contrary, the JME task shares conceptual similarities with embodied cognition theory. Beyond its social implications, another hypothesis posits that the JME arises from the joint cognitive representation of both actors involved.^62^ During the task, the participants spontaneously increase their attention to their partner, monitor their partner’s instructions and represent their partner’s body movements in order to predict and simulate what the other person is supposed to do. In line with this hypothesis, several studies^16,63-65^ have shown that when we want to recognize facial expressions, the emotional context of the surrounding scene plays a role. This context can be created by, for instance, telling participants highly pleasant or unpleasant stories to set a specific emotional tone before they are asked to identify emotional faces^64^ or by having emotional voice tones accompany the face recognition task.^65^ Similarly, an effect of social scene context has been shown to influence body expression recognition.^66^

Patients with Huntington’s disease experience difficulties to understand and convey their own body movements and those of others, affecting how they express emotions physically.^21,22,27,67^ While observed in both groups, our results indicate a proportional decrease in the SPE and the JME among individuals with Huntington’s disease compared with controls. This decline corresponds with expectations, as participants facing difficulties in representing both their own and others’ body movements and facial expressions would likely struggle in accurately monitoring their own and their partner’ actions, leading to poorer task performance. However, the framework of embodied cognition as an explanation for the JME was questioned by the study conducted by Wagner *et al*.^32^ In their research, the authors manipulated two crucial parameters in the joint memory task: performing the task with or without observing the partner’s actions and varying the physical distance between the participant and the partner, with participants informed of each other’s category. Their findings indicate that the JME emerged even without visual and auditory cues of the other’s response, but this effect declined with the physical proximity between the participant and the partner. This contradicts the predictions of embodied cognition theory and instead supports the idea of the genuine social nature of JME. Consistently, our MRI morphometric analyses did not reveal any significant correlations with key brain regions associated with embodied cognition.

Another plausible explanation for the relative decrease in both the SPE and JME in those with Huntington’s disease could be linked to overall and mnesic poorer cognitive performances. This may be explained easily by the mere nature of the task, engaging mnesic and executive processes that are classically impaired in Huntington’s disease.^68-71^ Interestingly, while the JME correlated with performances in all cognitive tasks, the SPE was only associated with performance in the Hopkins Verbal Learning test. This suggests that the SPE may represent a more resilient effect against cognitive and executive dysfunction, possibly due to the imperative nature of prioritizing self-information for ensuring proper cognitive functioning. Accordingly, empirical studies on self have consistently demonstrated a self-referential advantage across various cognitive domains, including attention, memory and action cultures and lifespan.^52^

Here, we lay the groundwork for a reflection on interpersonal interaction by approaching ecological conditions of interaction in contrast to classical models of social interaction such as ToM and embodied cognition. By purposefully linking selfishness to these classical social concepts through the use of the JME, we offer an additional, non-exclusive approach to assessing social interaction behaviours: the balance of attention allocation between oneself and others. Humans naturally pay attention to other group members to glean valuable social information about their identity, emotions and likely intentions.^1^ This approach enabled us to observe an engagement of participants with Huntington’s disease towards partner-relevant information without any prior cues or incentives. While traditional definitions of selfishness imply a conscious decision to prioritize one’s own interests over others,^7,8^ the demonstration of this spontaneous attention to the partner suggests an absence of self-centred bias, indicating that voluntary, ego-driven behaviour would be highly improbable in participants with Huntington’s disease.

## Conclusion

Our study aimed to explore social cognitive interaction in patients with Huntington’s disease within a more ecological setting than traditional ToM and embodied cognition tasks. Our findings challenge the stigma that individuals with Huntington’s disease are unable to pay attention to others, thereby demonstrating selfish behaviours. Instead, they displayed a spontaneous tendency to recall partner-relevant information, indicating a genuine interest in their partners. Preliminary morphometric MRI results suggest that this effect may be linked to the right orbitofrontal cortex. Although this social effect was present in participants with Huntington’s disease, it was less pronounced compared with controls, likely due to cognitive deficits in the former group. These findings suggest that individuals with Huntington’s disease may be able to improve their cognitive abilities by engaging in enriched daily interactions with their caregivers.

## Supplementary Material

fcae440_Supplementary_Data

## Data Availability

The anonymized data sets of the current study are available from the corresponding author on reasonable request.

## References

[1] A novel gene containing a trinucleotide repeat that is expanded and unstable on Huntington’s disease chromosomes . The Huntington’s Disease Collaborative Research Group. Cell. 1993;72(6):971–983. 8458085 10.1016/0092-8674(93)90585-e

[2] Bates GP , Dorsey R, Gusella JF, et al Huntington disease. Nat Rev Dis Primer. 2015;1:15005. 10.1038/nrdp.2015.5 27188817

[3] Boileau NR , Paulsen JS, Ready RE, Hahn EA, Lai JS, Carlozzi NE. Understanding domains that influence perceived stigma in individuals with Huntington disease. Rehabil Psychol. 2020;65(2):113–121. 31961169 10.1037/rep0000311 PMC7195240

[4] Wallace DC , Hall AC. Evidence of genetic heterogeneity in Huntington’s chorea. J Neurol Neurosurg Psychiatry. 1972;35(6):789–800. 4265114 10.1136/jnnp.35.6.789 PMC494182

[5] Snowden JS , Gibbons ZC, Blackshaw A, et al Social cognition in frontotemporal dementia and Huntington’s disease. Neuropsychologia. 2003;41(6):688–701. 12591026 10.1016/s0028-3932(02)00221-x

[6] Eddy CM , Parkinson EG, Rickards HE. Changes in mental state and behaviour in Huntington’s disease. Lancet Psychiatry. 2016;3(11):1079–1086. 27663851 10.1016/S2215-0366(16)30144-4

[7] Carlson RW , Adkins C, Crockett MJ, Clark MS. Psychological selfishness. Perspect Psychol Sci. 2022;17(5):1359–1380. 35436157 10.1177/17456916211045692

[8] Raine A , Uh S. The selfishness questionnaire: Egocentric, adaptive, and pathological forms of selfishness. J Pers Assess. 2019;101(5):503–514. 29671625 10.1080/00223891.2018.1455692

[9] Frimer JA , Schaefer NK, Oakes H. Moral actor, selfish agent. J Pers Soc Psychol. 2014;106(5):790–802. 24749822 10.1037/a0036040

[10] Rachlin H . Altruism and selfishness. Behav Brain Sci. 2002;25(2):239–250. 12744145 10.1017/s0140525x02000055

[11] Crocker J , Canevello A, Brown AA. Social motivation: Costs and benefits of selfishness and otherishness. Annu Rev Psychol. 2017;68(1):299–325. 27362501 10.1146/annurev-psych-010416-044145

[12] Brüne M , Blank K, Witthaus H, Saft C. “Theory of mind” is impaired in Huntington’s disease. Mov Disord Off J Mov Disord Soc. 2011;26(4):671–678. 10.1002/mds.23494 21384426

[13] Caillaud M , Desgranges B, Verny C, Allain P. Altération de la cognition sociale dans la maladie de Huntington: Neuropsychologie et neuro-imagerie, deux approches complémentaires. Rev Neuropsychol. 2015;7(2):109–116.

[14] Bora E , Velakoulis D, Walterfang M. Social cognition in Huntington’s disease: A meta-analysis. Behav Brain Res. 2016;297:131–140. 26455876 10.1016/j.bbr.2015.10.001

[15] Allain P , Havet-Thomassin V, Verny C, et al Evidence for deficits on different components of theory of mind in Huntington’s disease. Neuropsychology. 2011;25(6):741–751. 21728429 10.1037/a0024408

[16] Barrett LF , Lindquist KA, Gendron M. Language as context for the perception of emotion. Trends Cogn Sci. 2007;11(8):327–332. 17625952 10.1016/j.tics.2007.06.003 PMC2225544

[17] Palminteri S , Justo D, Jauffret C, et al Critical roles for anterior insula and dorsal striatum in punishment-based avoidance learning. Neuron. 2012;76(5):998–1009. 23217747 10.1016/j.neuron.2012.10.017

[18] Snowden JS . The neuropsychology of Huntington’s disease. Arch Clin Neuropsychol. 2017;32(7):876–887. 28961886 10.1093/arclin/acx086

[19] Wilson M . Six views of embodied cognition. Psychon Bull Rev. 2002;9(4):625–636. 12613670 10.3758/bf03196322

[20] Gallezot C , Riad R, Titeux H, et al Emotion expression through spoken language in Huntington disease. Cortex. 2022;155:150–161. 35986957 10.1016/j.cortex.2022.05.024

[21] de Gelder B , Van den Stock J, de Diego Balaguer R, Bachoud-Lévi AC. Huntington’s disease impairs recognition of angry and instrumental body language. Neuropsychologia. 2008;46(1):369–373. 18061217 10.1016/j.neuropsychologia.2007.10.015

[22] Van den Stock J , De Winter F, Ahmad R, et al Functional brain changes underlying irritability in premanifest Huntington’s disease. Hum Brain Mapp. 2015;36(7):2681–2690. 25858294 10.1002/hbm.22799 PMC6869704

[23] Henley SMD , Novak MJU, Frost C, King J, Tabrizi SJ, Warren JD. Emotion recognition in Huntington’s disease: A systematic review. Neurosci Biobehav Rev. 2012;36(1):237–253. 21699916 10.1016/j.neubiorev.2011.06.002

[24] Gallagher HL , Frith CD. Functional imaging of “theory of mind”. Trends Cogn Sci. 2003;7(2):77–83. 12584026 10.1016/s1364-6613(02)00025-6

[25] Abu-Akel A , Shamay-Tsoory S. Neuroanatomical and neurochemical bases of theory of mind. Neuropsychologia. 2011;49(11):2971–2984. 21803062 10.1016/j.neuropsychologia.2011.07.012

[26] Brink TT , Urton K, Held D, et al The role of orbitofrontal cortex in processing empathy stories in 4- to 8-year-old children. Front Psychol. 2011;2:80. 21687450 10.3389/fpsyg.2011.00080 PMC3110480

[27] Trinkler I , Devignevielle S, Achaibou A, et al Embodied emotion impairment in Huntington’s disease. Cortex. 2017;92:44–56. 28399433 10.1016/j.cortex.2017.02.019

[28] Gallotti M , Frith CD. Social cognition in the we-mode. Trends Cogn Sci. 2013;17(4):160–165. 23499335 10.1016/j.tics.2013.02.002

[29] Sebanz N , Knoblich G, Prinz W. Representing others’ actions: Just like one’s own? Cognition. 2003;88(3):B11–B21. 12804818 10.1016/s0010-0277(03)00043-x

[30] Böckler A , Knoblich G, Sebanz N. Effects of a coactor’s focus of attention on task performance. J Exp Psychol Hum Percept Perform. 2012;38(6):1404–1415. 22409143 10.1037/a0027523

[31] Elekes F , Sebanz N. Effects of a partner’s task on memory for content and source. Cognition. 2020;198:104221. 32058100 10.1016/j.cognition.2020.104221

[32] Wagner U , Giesen A, Knausenberger J, Echterhoff G. The joint action effect on memory as a social phenomenon: The role of cued attention and psychological distance. Front Psychol. 2017;8:1697. 29051742 10.3389/fpsyg.2017.01697 PMC5633604

[33] Eskenazi T , Doerrfeld A, Logan GD, Knoblich G, Sebanz N. Your words are my words: Effects of acting together on encoding. Q J Exp Psychol. 2013;66(5):1026–1034. 10.1080/17470218.2012.725058 23035698

[34] Sui J , He X, Humphreys GW. Perceptual effects of social salience: Evidence from self-prioritization effects on perceptual matching. J Exp Psychol Hum Percept Perform. 2012;38(5):1105–1117. 22963229 10.1037/a0029792

[35] Kampis D , Southgate V. Altercentric cognition: How others influence our cognitive processing. Trends Cogn Sci. 2020;24(11):945–959. 32981846 10.1016/j.tics.2020.09.003

[36] Penney JB , Vonsattel JP, MacDonald ME, Gusella JF, Myers RH. CAG repeat number governs the development rate of pathology in Huntington’s disease. Ann Neurol. 1997;41(5):689–692. 9153534 10.1002/ana.410410521

[37] Shoulson I . Huntington disease: Functional capacities in patients treated with neuroleptic and antidepressant drugs. Neurology. 1981;31(10):1333–1335. 6125919 10.1212/wnl.31.10.1333

[38] Mattis S. Mental Status Examination for organic mental syndrome in the elderly patients. In: Bellack L, Karusu TB, eds. *Geriatric psychiatry: A handbook for psychiatrists and primary care physicians*. Grune & Stratton; 1976:77-121.

[39] Unified Huntington’s Disease Rating Scale: Reliability and consistency. Huntington Study Group. Mov Disord. 1996;11(2):136–142. 8684382 10.1002/mds.870110204

[40] McNally G , Rickards H, Horton M, Craufurd D. Exploring the validity of the short version of the problem behaviours assessment (PBA-s) for Huntington’s disease: A Rasch analysis. J Huntingt Dis. 2015;4(4):347–369. 10.3233/JHD-150164 26756591

[41] Rieu D , Bachoud-Lévi AC, Laurent A, Jurion E, Barba GD. Adaptation française du « Hopkins verbal learning test ». Rev Neurol (Paris). 2006;162(6):721–728. 16840980 10.1016/s0035-3787(06)75069-x

[42] Brandt J . The Hopkins Verbal Learning Test: Development of a new memory test with six equivalent forms. Clin Neuropsychol. 1991;5:125–142.

[43] Schobel SA , Palermo G, Auinger P, et al Motor, cognitive, and functional declines contribute to a single progressive factor in early HD. Neurology. 2017;89(24):2495–2502. 29142089 10.1212/WNL.0000000000004743 PMC5729794

[44] Good CD , Johnsrude IS, Ashburner J, Henson RN, Friston KJ, Frackowiak RS. A voxel-based morphometric study of ageing in 465 normal adult human brains. NeuroImage. 2001;14(1 Pt 1):21–36. 11525331 10.1006/nimg.2001.0786

[45] Smith SM , Jenkinson M, Woolrich MW, et al Advances in functional and structural MR image analysis and implementation as FSL. NeuroImage. 2004;23(Suppl 1):S208–S219. 15501092 10.1016/j.neuroimage.2004.07.051

[46] Douaud G , Smith S, Jenkinson M, et al Anatomically related grey and white matter abnormalities in adolescent-onset schizophrenia. Brain J Neurol. 2007;130(Pt 9):2375–2386. 10.1093/brain/awm184 17698497

[47] Lunven M , Dominguez KH, Youssov K, et al A new approach to digitized cognitive monitoring: Validity of the SelfCog in Huntington’s disease. Brain Commun. 2023;5(2):fcad043. 10.1093/braincomms/fcad043 PMC10018460 36938527

[48] Wen T , Egner T. Retrieval context determines whether event boundaries impair or enhance temporal order memory. Cognition. 2022;225:105145. 35483158 10.1016/j.cognition.2022.105145

[49] Desebrock C , Spence C. The self-prioritization effect: Self-referential processing in movement highlights modulation at multiple stages. Atten Percept Psychophys. 2021;83(6):2656–2674. 33861428 10.3758/s13414-021-02295-0 PMC8302500

[50] Sui J , Rotshtein P. Self-prioritization and the attentional systems. Curr Opin Psychol. 2019;29:148–152. 30913475 10.1016/j.copsyc.2019.02.010

[51] Schäfer S , Wentura D, Frings C. Self-prioritization beyond perception. Exp Psychol. 2015;62(6):415–425. 27120563 10.1027/1618-3169/a000307

[52] Singh D , Karnick H. Self-prioritization effect in children and adults. Front Psychol. 2022;13:726230. 35783811 10.3389/fpsyg.2022.726230 PMC9244848

[53] Frings C , Wentura D. Self-priorization processes in action and perception. J Exp Psychol Hum Percept Perform. 2014;40(5):1737–1740. 24999614 10.1037/a0037376

[54] Mastromauro C , Myers RH, Berkman B, Opitz JM, Reynolds JF. Attitudes toward presymptomatic testing in Huntington disease. Am J Med Genet. 1987;26(2):271–282. 2949611 10.1002/ajmg.1320260205

[55] Mand CM , Gillam L, Duncan RE, Delatycki MB. “I’m scared of being like mum”: Th experience of adolescents living in families with Huntington disease. J Huntingt Dis. 2015;4(3):209–217. 10.3233/JHD-150148 26443924

[56] Wong S , Irish M, Leshikar ED, et al The self-reference effect in dementia: Differential involvement of cortical midline structures in Alzheimer’s disease and behavioural-variant frontotemporal dementia. Cortex. 2017;91:169–185. 27771044 10.1016/j.cortex.2016.09.013 PMC5760181

[57] Takagishi H , Koizumi M, Fujii T, Schug J, Kameshima S, Yamagishi T. The role of cognitive and emotional perspective taking in economic decision making in the ultimatum game. PLoS One. 2014;9(9):e108462. 25255309 10.1371/journal.pone.0108462 PMC4177993

[58] Cowell J , Samek A, List J, Decety J. The curious relation between theory of mind and sharing in preschool age children. PLoS One. 2015;10:e0117947. 25658696 10.1371/journal.pone.0117947 PMC4320030

[59] Mulvey KL , Buchheister K, McGrath K. Evaluations of intergroup resource allocations: The role of theory of mind. J Exp Child Psychol. 2016;142:203–211. 26525855 10.1016/j.jecp.2015.10.002

[60] Jonker FA , Jonker C, Scheltens P, Scherder EJA. The role of the orbitofrontal cortex in cognition and behavior. Rev Neurosci. 2015;26(1):1–11. 25252749 10.1515/revneuro-2014-0043

[61] Davis MH. *Interpersonal reactivity index (IRI)*. APA PsycTests. 1980.

[62] Wenke D , Atmaca S, Holländer A, Liepelt R, Bäss P, Prinz W. What is shared in joint action? Issues of co-representation, response conflict, and agent identification. Rev Philos Psychol. 2011;2:147–172.

[63] Meeren HKM , van Heijnsbergen CCRJ, de Gelder B. Rapid perceptual integration of facial expression and emotional body language. Proc Natl Acad Sci U S A. 2005;102(45):16518–16523. 16260734 10.1073/pnas.0507650102 PMC1283446

[64] Carroll JM , Russell JA. Do facial expressions signal specific emotions? Judging emotion from the face in context. J Pers Soc Psychol. 1996;70(2):205–218. 8636880 10.1037//0022-3514.70.2.205

[65] Gelder B , Vroomen J. The perception of emotions by ear and eye. Cogn Emot. 2000;14:289–311.

[66] Kret ME , de Gelder B. Social context influences recognition of bodily expressions. Exp Brain Res. 2010;203(1):169–180. 20401473 10.1007/s00221-010-2220-8 PMC2862946

[67] Trinkler I , de Langavant LC, Bachoud-Lévi AC. Joint recognition-expression impairment of facial emotions in Huntington’s disease despite intact understanding of feelings. Cortex. 2013;49(2):549–558. 22244587 10.1016/j.cortex.2011.12.003

[68] Lawrence AD , Sahakian BJ, Hodges JR, Rosser AE, Lange KW, Robbins TW. Executive and mnemonic functions in early Huntington’s disease. Brain. 1996;119(5):1633–1645. 8931586 10.1093/brain/119.5.1633

[69] Peinemann A , Schuller S, Pohl C, Jahn T, Weindl A, Kassubek J. Executive dysfunction in early stages of Huntington’s disease is associated with striatal and insular atrophy: A neuropsychological and voxel-based morphometric study. J Neurol Sci. 2005;239(1):11–19. 16185716 10.1016/j.jns.2005.07.007

[70] Snowden JS , Craufurd D, Thompson J, Neary D. Psychomotor, executive, and memory function in preclinical Huntington’s disease. J Clin Exp Neuropsychol. 2002;24(2):133–145. 11992196 10.1076/jcen.24.2.133.998

[71] de Langavant LC , Fénelon G, Benisty S, Boissé MF, Jacquemot C, Bachoud-Lévi AC. Awareness of memory deficits in early stage Huntington’s disease. PLoS One. 2013;8(4):e61676. 23620779 10.1371/journal.pone.0061676 PMC3631142

