# Supplementary material for: The joint memory effect: challenging the selfish stigma in Huntington’s disease?
Source: Brain Commun. 2024 Dec 9;7(1):fcae440. doi: 10.1093/braincomms/fcae440 (PMC11693399; doi:10.1093/braincomms/fcae440)

Supplementary Materials

Supplementary Table 1. Words used in the Shared Memory Task according to the semantic categories (Animals, Fruits/Vegetables and Manufactured Objects) and the language of the participants (French, English and German).

Category	French	English	German
Animals			
	antilope	antelope	antilope
	baleine	whale	wal
	brebis	sheep	schaf
	cachalot	**jellyfish***	pottwal
	caribou	caribou	karibu
	castor	beaver	biber
	chameau	camel	camel
	chimpanzé	chimpanzee	schimpanse
	coccinelle	ladybug	marienkäfer
	cochon	pig	schwein
	colibri	hummingbird	kolibri
	corbeau	raven	rab
	crocodile	crocodile	krocodil
	écrevisse	crayfish	flusskrebs
	écureuil	squirrel	eichhörnchen
	grenouille	frog	frosch
	hérisson	hedgehog	igel
	iguane	iguana	leguan
	koala	koala	koala
	lémurien	lemur	lemur
	lézard	lizard	eidechse
	libellule	dragonfly	libelle
	panda	panda	panda
	panthère	panther	panther
	papillon	butterfly	schmetterling
	perroquet	parrot	papagei
	renard	fox	fuchs
	requin	shark	hai
	scarabée	ladybug	käfer
	taureau	bull	stier
	tortue	tortle	schildkröte
	vipère	viper	viper
Fruits/Vegetables			
	ananas	pineapple	ananas
	artichaut	artichoke	artishocke
	aubergine	eggplant	aubergine
	avocat	avocado	avocado
	brocoli	broccoli	brokkoli

	cassis	blackcurrant	schwarze johannisbeere
	céleri	celery	sellerie
	châtaigne	**carrot***	kastanie
	ciboulette	chive	schnittlauch
	cornichon	gherkin	gurke
	cresson	**cherry***	kresse
	endive	endive	endivie
	goyave	guaya	guave
	groseille	currant	johannisbeere
	haricot	bean	bohne
	mandarine	mandarin	mandarine
	melon	melon	melone
	mirabelle	**banana***	mirabelle
	navet	turnip	rübe
	nectarine	nectarine	nektarine
	noisette	hazelnut	haselnuss
	oignon	onion	zwiebel
	olive	olive	olive
	pamplemousse	grapefruit	grapefruit
	pastèque	watermelon	wassermelone
	poivron	pepper	paprika
	pomelo	pomelo	pomelo
	potiron	pumpkin	kürbis
	pruneau	prune	pflaume
	radis	radish	rettich
	rhubarbe	rhubarbe	rhabarber
	salsifis	**pear***	schwarzwurzeln
Manufactured Objects			
	agrafeuse	stapler	tacker
	ballon	balloon	ballon
	bocal	jar	glas
	bougie	candle	kerze
	boussole	compass	kompass
	brouette	wheelbarrow	schubkarre
	carafe	carafe	karaffe
	cendrier	ashtray	aschenbecher
	ciseau	chisel	meißel
	compas	**handcuffs***	zirkel
	cuillère	spoon	löffel
	domino	domino	domino
	écouteur	headphones	hörer
	entonnoir	funnel	trichter
	étagère	shelf	regal
	éventail	**guitar***	fächer
	fourchette	fork	gabel
	lavabo	washbassin	waschbecken
	oreiller	pillow	kissen
	panier	basket	korb
			sieb

	passoire	strainer	geldbeutel
	portefeuille	wallet	winkelmesser
	rapporteur	protractor	schläger
	raquette	racket	schüssel
	saladier	bowl	gartenschere
	sécateur	**pestle***	kugelschreiber
	stylo	**pencil***	hocker
	tabouret	stool	thermometer
	thermomètre	thermometer	mäher
	tondeuse	lawn-mower	trampolin
	trampoline	trampoline	dreirad
	tricycle	tricycle	

English and German words were translated from the French list. Words with * have been change due to translation problems (important difference in syllable count, lexical frequency, …)

Supplementary Table 2. Response time (msec, means ± standard deviations) and accuracy at the encoding phase

	**Controls**		**Huntington's Disease participants**	
	Alone	In Pair	Alone	In Pair
Response Time (RT)	1065 ± 213	1000 ± 237	1390 ± 323	1268 ± 335
Accuracy	97.43 ± 3.58	98.44 ± 2.53	94.84 ± 4.79	96.20 ± 4.67

Supplementary Figure 1: Interaction between social value (Irrelevant, Self) and condition (Alone, Pair) across both groups (Huntington's Disease, Controls)

We performed analyses of variance (ANOVA) on the number of recalled words with group (n=56 Control, n=69 Huntington's disease participants), social value (self-words, irrelevant words), condition (Alone, Pair) and their interaction as independent factors. The interaction between condition and social value was significant ($F(1,492)=42.24$, $p<0.001$) and represented in this Figure. The triple interaction was not significant ($F(1,492)=2.55$, $p=0.11$). Tukey comparisons of means showed that in the Alone and in the Pair conditions, participants recalled more self-words than irrelevant ones (respectively: difference = 0.42, $p < 0.001$; difference = 1.01, $p < 0.001$). Participants recalled more self-words in the Pair condition compared to the Alone condition (difference = 0.60, $p < 0.001$). This latter difference was not observed for the irrelevant words (difference = 0.003, $p = 0.99$).

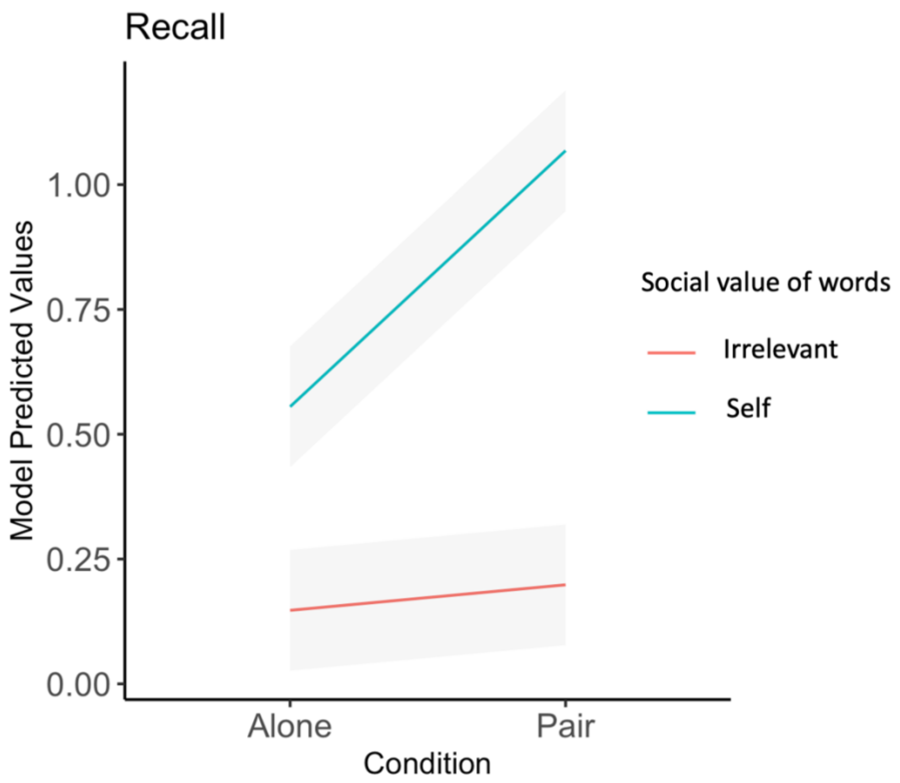

Supplementary Figure 2: Triple interaction between social value (Irrelevant, Self) and condition (Alone, Pair) and groups (Huntington's Disease, Controls)

We performed analyses of variance (ANOVA) on the number of recalled words with group (n=56 Controls, n=69 Huntington's disease participants), social-value (partner-words, irrelevant words), and condition (Alone, Pair) and their interaction as independent factors. The triple interaction was significant and illustrated in this Figure. ($F(1, 492)=4.34$, $p = 0.038$). Tukey post-hoc analysis confirmed the presence of a Joint Memory Effect in both groups by a higher recall of partner- words than of irrelevant words in both groups in the Pair condition (Controls: difference = 0.45, $p < 0.001$; Huntington's disease participants: difference = 0.34, $p < 0.001$). The recall of partner-words increased in the Pair condition compared to the Alone condition in both groups (Controls: difference = 0.51, $p < 0.001$; Huntington's disease participants: difference = 0.27, $p < 0.01$) in contrast with the recall of irrelevant words that did not changed between conditions (Controls: difference = -0.06, $p > 0.99$; Huntington's disease participants: difference = 0.05, $p > 0.99$).

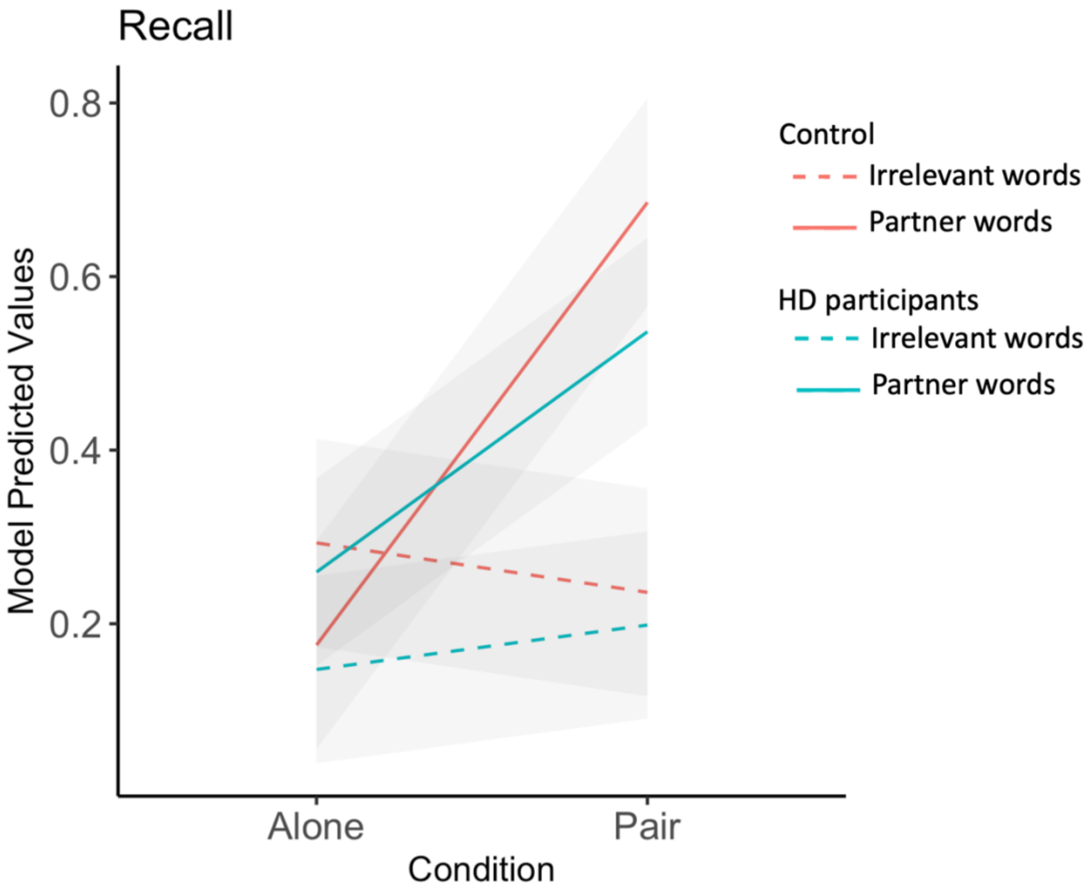

Supplement: fcae440_Supplementary_Data [file fcae440_supplementary_data.pdf]
